# MTHFR polymorphisms and vitamin B12 deficiency: correlation between mthfr polymorphisms and clinical and laboratory findings

**DOI:** 10.1007/s00277-024-05937-z

**Published:** 2024-08-28

**Authors:** Sabrina Giammarco, Patrizia Chiusolo, Roberto Maggi, Monica Rossi, Gessica Minnella, Elisabetta Metafuni, Francesco D’Alò, Simona Sica

**Affiliations:** 1 Dipartimento di Scienze di Laboratorio ed Infettivologiche, Fondazione Policlinico Universitario A. Gemelli IRCCS, Rome, Italy; 2 Sezione di Ematologia, Dipartimento di Scienze Radiologiche ed Ematologiche, Università Cattolica del Sacro Cuore, Rome, Italy; 3 Fondazione Policlinico Universitario “A. Gemelli” -IRCCS, Rome, Italy

**Keywords:** MTHFR, Polymorphism, Cobalamin deficiency, Gastritis, Anemia

## Abstract

Vitamin B12 deficiency is a common condition that causes a variety of disorders ranging from the development of megaloblastic anemia to the building up of neurological damage. Historically one of the leading causes of B12 deficiency appears to be secondary to malabsorption in part caused by the development of atrophic gastritis in pernicious anemia. More recently B12 deficiency could also depend on dietary restrictions. Cobalamin deficiency also appears to be closely related to folate metabolism, causing a reduction in methionine synthase activity. This results in the accumulation of 5-methyltetrahydrofolate (5-MTHF) and defective DNA synthesis. It has been hypothesized that reduced activity of the enzyme methylene-tetrahydrofolate reductase (MTHFR) could reduce the production of 5-MTHF, thereby shifting folate metabolism to thymidylate synthesis and promoting proper DNA synthesis. Our aim was to investigate the role of the C677T and A1298C MTHFR gene polymorphisms, which are associated with reduced enzyme activity, in predisposing to the development of anemia, neurological symptoms, and atrophic gastritis in a population of 105 consecutive Italian patients with cobalamin deficiency. We found statistically significant correlations between the degree of anemia and thrombocytopenia and the C677T MTHFR polymorphism, while hemoglobin levels alone significantly correlated with A1298C polymorphism, contradicting the potential protective role of these polymorphisms. Furthermore, in patients with atrophic gastritis, we found an association between the absence of parietal cell antibodies and the presence of the C677T polymorphism in homozygosity. Our results suggest a role for MTHFR enzyme activity in the severity of hematologic manifestations of vitamin B12 deficiency and as an independent mechanism of predisposition to the development of atrophic gastritis.

## Introduction

Vitamin B_12_ deficiency is a common condition and it represents a significant public health problem, particularly among the elderly. Vitamin B_12_ (cobalamin) is a cofactor for only two enzymes: methionine synthase and L- methyl-malonyl-coenzyme A mutase. Uptake in the gastrointestinal tract depends on intrinsic factor, which is synthesized by gastric parietal cells, and on the “cubam receptor” in the distal ileum [1]. The most common cause of B_12_ deficiency is believed to be B_12_ malabsorption caused by chronic gastritis, gastric atrophy, the use of proton pump inhibitors, and low intake due to long-term vegetarian or vegan dietary [2].

The classical pathophysiological manifestations of B_12_ deficiency include megaloblastic anemia, neuropathy, hyperhomocysteinemia, and deterioration of cognitive function.

Interaction between folate and B12 causes impaired DNA biosynthesis leading to ineffective erythropoiesis, intramedullary hemolysis, release of lactate dehydrogenase, and megaloblastic anemia seen in both vitamin deficiencies [1].

Vitamin B12 plays a key role in the myelination process of the central nervous system. The specific type of neuropathy in vitamin B_12_ deficiency is most likely the result of hypomethylation of myelic basic protein. This can lead to sequelae such as peripheral neuropathy, depression, cognitive disturbances, and dementia [3].

Moreover, decreased methionine synthase activity causes impaired regeneration of methionine from homocysteine, resulting in homocysteine accumulation, which may contribute to the risk of vascular disease [4].

Folate metabolism plays an essential role in DNA synthesis and methylation processes. The methylfolate trap hypothesis assumes that 5-methyltetrahydrofolate (5-MTHF) cannot be transformed back to its precursor 5,10-methylenetetrahydrofolate (5,10-MTHF), because the reaction catalyzed by MTHFR is irreversible. In cobalamin deficiency, methionine synthase is inactive, causing the accumulation of 5-MTHF, which is a poor substrate for folypolyglutamate synthase. The C677T and A1298C MTHFR gene polymorphisms are associated with decreased enzyme activity. Thus, the homozygous genotype may protect patients with reduced methionine synthase activity from defective DNA synthesis because folate metabolism tends to be shifted to thymidylate synthesis.

Several studies have reported an association between disturbances of folate metabolism and increased risk of gastric cancer. Moreover, intestinal metaplasia, which is a frequent gastric complication of atrophic gastritis associated with vitamin B_12_ deficiency, is a risk factor for adenocarcinoma. [5, 6].

We aimed to examine cobalamin-deficient patients with reduced MTHFR activity, according to above mentioned MTHFR polymorphisms, to evaluate their predisposition to develop anemia and its severity, neurological symptoms, and atrophic gastritis.

## Materials and methods

We enrolled 105 Italian patients (53 males and 52 females), with a median age of 58 years (range 19–90), who were consecutively admitted to our Hematology Division from January 2006 to December 2022, with a diagnosis of megaloblastic anemia, with clinical and biochemical evidence of cobalamin deficiency. The study was conducted according to the Declaration of Helsinki and was approved by the Internal Review Board of the Hematology Department.

At diagnosis, the hemoglobin median value was 7.3 g/dl for men (range: 3.1–13.3 g/dl) and 6.8 g/dl for women (range: 3.2–14.3 g/dl). Mean corpuscular volume (MCV) median value was 108 fl. (range: 77–138). Median white blood count was 3.9 × 10^9^/l (range: 1.16-13.19 × 10^9^/l). Median polymorphonuclear (PMN) count was 2.16 × 10^9^/l (range: 0.41-9.8 × 109/l). Median platelets count was 97 × 10^9^/l (range 14-460 × 10^9^/l).

The median plasmatic vitamin B_12_ dosage by CLIA was 74 pg/ml (range: 9-367 pg/ml; normal values 187–883 pg/ml). The median plasmatic folate dosage was 6.0 ng/ml (range: 0.7–41 ng/ml; normal values: >4 ng/ml). The median plasmatic ferritin dosage was 191 ng/ml (range: 4-1600 ng/ml; normal values: 12–290 ng/ml). Anti-parietal cell antibodies (PCA) were available in 88 out of 106 patients.

Patients’ characteristics are summarized in Table 1.

**Table 1:** Patients’ characteristics

Patients (*n*)	105
M/F	53/52
Median age; years (range)	58; (19–90)
Median hemoglobin value (g/dl); (range)	7; (3.1–14.3)
Median MCV value (fl.); (range)	108; (77–138)
Median PMN value (x10^9^/L); (range)	3.9; (1.16–13.19)
Median platelets value (x10^9^/L); (range)	97; (14–460)
Atrophic gastritis	52
PCA positivity	22
Neurological symptoms	7

Peripheral blood samples were obtained after informed consent from each patient or guardian for use in biological studies. All patients were tested for C677T and A1298C SNPS by polymerase chain reaction (PCR) techniques, as previously described [7, 8].

Statistical analysis was performed using Fisher’s exact test and x^2^ test for categorical variables and Kruskal Wallis test and Mann-Whitney test for continuous variables.

The significance level was set at *p* < 0.05.

## Results

By univariate analysis, we correlate hemoglobin value, MCV, white blood count (WBC), neutrophil count, platelets count, folate and B12 levels, neurological symptoms, and presence of atrophic gastritis and positivity of parietal cells antibody (PCA) at diagnosis with the distribution of C677T and A1298C genotypes. The following prevalence of the different genotypes was observed: for MTHFR C677T, 32 patients (30%) were wild type (CC), 49 (47%) had heterozygous genotype (CT) and 24 (23%) were homozygous (TT); for MTHFR A1298C, 53 patients (50%) were wild type (AA), 46 (43%) had heterozygous genotype (AC) and 4 (7%) were homozygous (CC). The distribution of the three polymorphisms is quite similar to those reported in the Italian healthy controls from our previous study on MTHFR genotypes [9].

We found a statistically significant correlation between patients carrying MTHFR C677T homozygous and heterozygous variant and lower hemoglobin value (*p* = 0.01) and lower platelet count (*p* = 0.006). We found a trend that did not reach statistical significance between patients carrying MTHFR C677T homozygous and heterozygous variants and lower PMN count (*p* = 0.06).

We found a trend of higher ferritin value (*p* = 0.05) between patients carrying MTHFR C677T homozygous and heterozygous variants.

Regarding MTHFR A1298C polymorphism, we found a statistical correlation between homozygous and heterozygous variants and lower hemoglobin values (*p* = 0.02).

We found no statistical correlation between MTHFR polymorphisms and B12 and folate levels, LDH level, spleen size, neurological symptoms, and gastropathy.

All seven patients with neurological symptoms presented primarily with peripheral neuropathy; they all had very low levels of vitamin b12, below 100 pg/ml. In patients with neurological symptoms, the distribution of the polymorphisms was as follows: for MTHFR C677T, 1 patient was wild type (CC), five patients had heterozygous genotype (CT) and 1 patient was homozygous (TT); for MTHFR A1298C, three patients were wild type (AA) and four patients had heterozygous genotype (AC).

Thirty-three out of 88 patients (37.5%) were positive for anti-parietal cell antibodies (PCA).

Fifty-two of the 105 patients (49%) had a diagnosis of gastropathy documented by endoscopy and histological examination. Among patients homozygous for MTHFR C677T the incidence of gastropathy was 62.5%. Interestingly we found a statistically significant association between patients carrying the MTHFR C677T homozygous genotype and the absence of anti-parietal antibodies (*p* = 0.03) despite a high incidence of gastropathy. Among patients with gastropathy, the analysis of plasma levels of folate showed the presence of low folate levels in patients with PCA negativity (*p* = 0.004).

Results are summarized in Table 2.

**Table 2:** Clinical and laboratory findings according to genotype frequencies

	MTHFR C677T				MTHFR A1298C *n* (%)			
	CC *n*(%)	CT *n*(%)	TT *n* (%)	*p*	AC *n*(%)	CC *n*(%)	GG *n*(%)	*p*
Anemia (83)	25 (30)	35 (41)	23 (29)	*0.01*	47 (58)	31 (38)	3 (4)	*0.02*
Thrombocytopenia (60)	19 (31)	25 (42)	16 (27)	*0.006*	31 (50)	26 (49)	1 (1)	*ns*
Neutropenia (28)	10 (35)	10 (35)	8 (30)	*ns*	16 (59)	11 (41)	0 (0)	*ns*
Mean corpuscular volume (79)	23 (29)	38 (48)	18 (23)	*ns*	39 (50)	34 (45)	4 (5)	*ns*
Atrophic gastritis (52)	15 (29)	22(42)	15 (29)	*ns*	30 (59)	21 (41)	0 (0)	*ns*
PCA negativity (23)	4 (17)	9 (40)	10 (43)	*0.03*	13 (45)	9 (55)	0 (0)	*ns*

## Discussion

Over time, MTHFR gene polymorphisms have been associated with a variety of conditions, including certain cancers, cardiovascular risk factors, neural tube defects, and vitamin B12 deficiency.

All of these studies have often produced conflicting results, especially about cancer susceptibility. This is mainly due to the different distribution of polymorphisms in different ethnic groups and the variability in lifestyle and dietary habits of different populations.

Associations between MTHFR gene polymorphisms and susceptibility to vitamin B12 deficiency have been described in the literature. Indeed, several studies have found an increased prevalence of the C677T polymorphism in homozygosity in affected patients compared with healthy controls in the Jordanian and Israeli populations [10, 11].

Another study conducted in pregnant women in South India showed an association of both polymorphisms investigated with the finding of B12 deficiency [12].

In our study, carried out in a population with established vitamin deficiency, the frequencies of the different polymorphisms overlapped with those of the general Italian population. This finding, in apparent contradiction with reported studies, could be due to several factors. First of all, we performed the study in a small sample size population, then we had to take into account the important multifactorial nature of this disease and the obvious differences between the different ethnic groups.

However, it is interesting to note that in our patients’ population, not only both polymorphisms do not prevent the development of anemia, as previously hypothesized, but they also seem to be associated with a more severe hematologic manifestation of vitamin B12 deficiency. It is likely that in individuals already more prone to vitamin deficiency, reduced MTHFR enzyme function leads to a metabolic imbalance, exacerbating anemia.

We found no statistical correlation between MTHFR polymorphisms and B12 and folate levels; this may be because some patients started folate therapy before coming to our attention; on the other hand, all patients had a vitamin B12 level below the lower limit. In particular patients with neurological symptoms presented very low levels of vitamin b12, confirming the role of myelination of the central nervous system as well as for the maintenance of its normal function, No correlation with polymorphisms’ distribution was found, due to the small number of patients with neurological symptoms.

More interesting, however, are the data regarding the gastropathy found in these patients and the presence or absence of gastric parietal cell antibodies. The role of MTHFR gene polymorphisms in the development of gastric cancer has long been debated. It is difficult to reach an unequivocal result in this regard, considering that the pathogenesis of cancer is multifactorial [13].

In the development of gastric cancer, it has been hypothesized that polymorphisms in this gene may indeed contribute to a predisposition to the development of gastropathy with associated intestinal metaplasia, which may then act as a precancerous lesion and increase the likelihood of later cancer development. [14].

Along these lines, recent studies have shown that the C677T polymorphism, in particular, is associated not only with an increased incidence of gastritis in Helicobacter pylori-negative individuals but also with a worse clinical course and a higher rate of malignant evolution in HP-mediated gastritis. [15, 16].

In our study, it is very interesting to note that in subjects who underwent endoscopic examination and were found to have gastropathy, an association was found between the C677T polymorphism of MTHFR and the absence of anti-gastric parietal cell antibodies. This finding, consistent with what has been described in the literature, would suggest a role for this polymorphism in predisposing to the development of gastric damage, in a manner completely independent of the autoimmune stimulus typical of pernicious anemia. Other suggestive data in this regard are the significantly lower serum folate levels in PCA-negative patients, suggesting the predominant role of metabolic patterns in this type of situation. However, none of these considerations can be applied to the A1298C polymorphism.

A recent study in the Chinese population demonstrated an improvement in intestinal metaplasia with folic acid supplementation in patients with chronic atrophic gastropathy, with a greater effect in patients with the C677T polymorphism in homozygosity, further confirming the above. [17].

In conclusion, our results suggest that the MTHFR C677T polymorphism, which directs folate toward thymidylate synthesis and away from methionine synthesis, does not protect against anemia and is associated with lower platelet counts.

It also suggests a possible correlation between MTHFR gene polymorphisms, folic acid levels, and the development of chronic atrophic gastritis.

Larger population studies may better elucidate the impact of these polymorphisms on the hematologic manifestations of B12 deficiency and the gastric complications that have the greatest impact on the prognosis of these patients.

## Data Availability

No datasets were generated or analysed during the current study.

## References

[1] Stabler SP (2013) Clinical practice. Vitamin B12 deficiency. N Engl J Med 368(2):149–160 23301732 10.1056/NEJMcp1113996

[2] Lindenbaum J, Healton EB, Savage DG, Brust JC, Garrett TJ, Podell ER et al (1988) Neuropsychiatric disorders caused by cobalamin deficiency in the absence of anemia or macrocytosis. N Engl J Med 318(26):1720–1728 3374544 10.1056/NEJM198806303182604

[3] Carmel R, Green R, Rosenblatt DS, Watkins D (2003) Update on cobalamin, folate, and homocysteine. Hematol Am Soc Hematol Educ Program. :62–81 10.1182/asheducation-2003.1.62 14633777

[4] Smulders YM, Blom HJ (2011) The homocysteine controversy. J Inherit Metab Dis 34(1):93–99 20567905 10.1007/s10545-010-9151-1 PMC3026670

[5] Vollset SE, Igland J, Jenab M, Fredriksen A, Meyer K, Eussen S et al (2007) The association of gastric cancer risk with plasma folate, cobalamin, and methylenetetrahydrofolate reductase polymorphisms in the European prospective investigation into Cancer and Nutrition. Cancer Epidemiol Biomarkers Prev 16(11):2416–2424 18006931 10.1158/1055-9965.EPI-07-0256

[6] Götze T, Röcken C, Röhl FW, Wex T, Hoffmann J, Westphal S et al (2007) Gene polymorphisms of folate metabolizing enzymes and the risk of gastric cancer. Cancer Lett 251(2):228–236 17208363 10.1016/j.canlet.2006.11.021

[7] Frosst P, Blom HJ, Milos R, Goyette P, Sheppard CA, Matthews RG et al (1995) A candidate genetic risk factor for vascular disease: a common mutation in methylenetetrahydrofolate reductase. Nat Genet 10(1):111–113 7647779 10.1038/ng0595-111

[8] Weisberg I, Tran P, Christensen B, Sibani S, Rozen R (1998) A second genetic polymorphism in methylenetetrahydrofolate reductase (MTHFR) associated with decreased enzyme activity. Mol Genet Metab 64(3):169–172 9719624 10.1006/mgme.1998.2714

[9] Chiusolo P, Reddiconto G, Cimino G, Sica S, Fiorini A, Farina G et al (2004) Methylenetetrahydrofolate reductase genotypes do not play a role in acute lymphoblastic leukemia pathogenesis in the Italian population. Haematologica 89(2):139–144 PMID: 15003888 15003888

[10] Al-Batayneh KM, Zoubi MSA, Shehab M, Al-Trad B, Bodoor K, Khateeb WA et al (2018) Association between MTHFR 677C > T polymorphism and vitamin B12 Deficiency: a case-control study. J Med Biochem 37(2):141–147 30581350 10.1515/jomb-2017-0051 PMC6294092

[11] Shiran A, Remer E, Asmer I, Karkabi B, Zittan E, Cassel A (2015) Association of Vitamin B12 Deficiency with homozygosity of the TT MTHFR C677T genotype, hyperhomocysteinemia, and endothelial cell dysfunction. Isr Med Assoc J 17(5):288–292 PMID: 26137654 26137654

[12] Barney AM, Danda S, Cherian AG, Aronraj J, Jayaprakash L, Abraham VJ et al (2022) Association of methylenetetrahydrofolate reductase *(MTHFR)* gene polymorphisms with vitamin B12 deficiency and adverse perinatal outcomes among pregnant women of rural South India - a cross sectional longitudinal study. J Perinat Med 50(9):1230–1238 35822733 10.1515/jpm-2022-0119

[13] Petrone I, Bernardo PS, Dos Santos EC, Abdelhay E, C677T (2021) and A1298C Polymorphisms in Breast Cancer, Gliomas and Gastric Cancer: A Review. Genes (Basel). ;12(4):587 10.3390/genes12040587 PMC8073588 33920562

[14] Palladino M, Chiusolo P, Reddiconto G, Marietti S, De Ritis D, Leone G, Sica S (2009) MTHFR polymorphisms involved in vitamin B12 deficiency associated with atrophic gastritis. Biochem Genet 47(9–10):645–650 19521764 10.1007/s10528-009-9256-0

[15] Saberi S, Zendehdel K, Jahangiri S, Talebkhan Y, Abdirad A, Mohajerani N et al (2012) Impact of methylenetetrahydrofolate reductase C677T polymorphism on the risk of gastric cancer and its interaction with Helicobacter pylori infection. Iran Biomed J 16(4):179–184 23183616 10.6091/ibj.1102.2012 PMC3600959

[16] Kong S, Ye F, Dang Y, Hua Y, Zhang G (2020) Association of MTHFR C677T polymorphism with severity and localization of chronic atrophic gastritis patients without Helicobacter pylori infection: a case control study. BMC Cancer 20(1):725 32758174 10.1186/s12885-020-07208-2 PMC7405366

[17] Kong S, Zhang G, Yang Z, Kong Z, Ye F (2023) Effects of folic acid supplementation on chronic atrophic gastritis based on MTHFR C677T polymorphism. Med (Baltim) 102(24):e33980 10.1097/MD.0000000000033980 PMC10270466 37327296

